# Needle gauge and length effects on human stem cell viability during injection: a comprehensive review

**DOI:** 10.3389/fcell.2026.1845972

**Published:** 2026-06-02

**Authors:** Umm E. Habiba, Sabiha Shamim, David Lawrence Greene

**Affiliations:** 1 Research and Development (R&D) Department, R3 Medical Research LLC, Scottsdale, AZ, United States; 2 Research and Development (R&D) Department, R3 Stem Cell LLC, Scottsdale, AZ, United States; 3 Research and Development, R3 Medical Research, Islamabad, Pakistan

**Keywords:** apoptosis, cell viability, cellular therapies, gauge, injection

## Abstract

The therapeutic efficacy of stem cell-based treatments depends critically on maintaining high cell viability through the injection process. Mechanical stress induced on cells during passage through injection needles represents a significant source of cell damage that can compromise therapeutic outcomes. This review examines the current understanding of how needle gauge (diameter) and length affect human stem cell viability, exploring the mechanical forces involved, empirical evidence across different stem cell types, and practical implications for clinical protocols. We analyze the competing demands of minimizing tissue trauma through smaller needles while preserving cell viability and provide evidence-based recommendations for optimizing injection parameters across various clinical applications.

## 1 Introduction

Stem cell therapies have emerged as promising treatments for numerous conditions, including orthopedic injuries, neurological disorders, cardiovascular disease, and neural conditions. The success of these interventions depends not only on the intrinsic properties of the stem cells but also on maintaining their viability throughout the administration process. Recent estimates suggest that cell death during injection can go above 95%, depending on injection parameters, representing a substantial loss of therapeutic potential (Hu et al., 2021).

During injection, cells experience multiple forms of mechanical stress. These include shear stress (generated as cells move through the narrow needle lumen at high velocity), extensional stress (occurs as cells elongate to pass through constrictions), collision forces (result from cell-wall and cell-cell interactions), and pressure gradients (created by the force applied to the syringe plunger) (Kharbikar et al., 2022). These forces can harm cell membranes and intracellular organelles, cause cell death, and reduce functional capacity (proliferation, differentiation capacity, and paracrine signaling) even in cells that remain technically viable.

**FIGURE 1:**
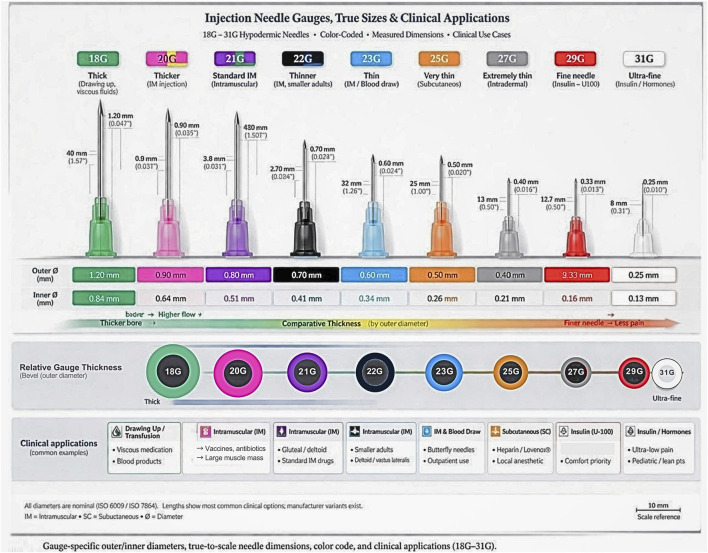
This figure illustrates commonly utilized hypodermic needle gauges in cellular therapy administration with standard sizes of conventional needle gauges.

Before the needle is selected for administration, several factors need to be considered. These include the gauge, length, shape of the bevel, location of administration and among other factors. Needle gauge follows an inverse relationship with diameter (higher gauge = smaller diameter); Large bore: 14G-18G (1.60–1.27 mm), Standard: 20G-23G (0.90–0.64 mm), Small bore: 25G-27G (0.51–0.41 mm) and Ultra-fine: 30G-32G (0.31–0.23 mm) (Morland, 2025) (Figure 1). Manufacturers typically cite longer needles to be ideal for intramuscular administration and shorter ones for subcutaneous.

Innovations like microneedles have been around for a while. However, in recent years, improvements in design have enhanced their use in regenerative medicine (Xu et al., 2021). They offer convenient, non-invasive, painless, and flexible, but precise applicability, with multifunctional control. They can be modified with photosensitizers or photothermal therapy. These sensors can extract information from interstitial fluid in the skin and other biochemical/electronic signals (Wang et al., 2023). Latest preclinical research explored hyaluronic acid methacryloyl microneedles to deliver human mesenchymal stem cell-derived exosomes in second-degree burns (Elakkawi et al., 2026). Their benefits have made them a popular topic of research for cases involving diabetic wounds (Cao et al., 2024), skin issues (Kumar et al., 2026), hair loss (Sun et al., 2026) and neurological disorders (Jeyavelkumaran et al., 2026).

This paper synthesizes evidence from *in vitro* studies, computational modeling, and clinical observations to provide a comprehensive understanding of needle-induced cell damage. A special focus was placed on human stem cells, including mesenchymal stem cells (MSCs) and hematopoietic stem cells, and induced pluripotent stem cells (iPSCs), examining how needle parameters should be optimized for different cell types and clinical applications.

## 2 Fluid dynamics of cell injection

The flow of cell suspensions through needles can be approximated using principles of fluid mechanics. The shear rate (γ) within a needle is given by γ = 4Q/(πr^3^); where Q is the volumetric flow rate, and r is the needle radius (Aguado et al., 2012). The shear stress (τ) experienced by cells depends on both the shear rate and the apparent viscosity (η) of the cell suspension (τ = ηγ). According to Awonusi et al., the highest shear stresses occur at the needle wall, with cells moving through the center experiencing lower stress levels (Awonusi et al., 2023).

## 3 Cell damage mechanisms

When shear stresses exceed the tensile strength of the lipid bilayer (typically 1–10 mN/m), the membrane ruptures, leading to immediate cell death or delayed apoptosis due to loss of membrane integrity (Suwannaphan et al., 2025). Sublethal mechanical stress activates mechanosensitive ion channels and signaling pathways, potentially affecting cell differentiation, proliferation, and secretory function even when viability appears preserved. Research has shown that MSCs subjected to moderate shear stress exhibit altered gene expression patterns and modified secretome profiles that persist for days after the mechanical insult. The cellular response to mechanical stress requires ATP for membrane repair and stress response activation, potentially depleting energy reserves needed for engraftment and therapeutic function (Dsouza et al., 2022). This metabolic burden may explain why some cells that survive injection show reduced proliferative capacity or altered differentiation potential.

Research across multiple cell types suggests critical shear stress thresholds ranging from less than 50 Pa (Minimal damage for most cell types) to more than 1,000 Pa (Substantial damage across most cell types) (Neves et al., 2025). These thresholds vary somewhat by cell type, with suspended hematopoietic cells generally more resistant than hybridoma cells forced into suspension, the vial size, and cell density.

## 4 Cell type variations

### 4.1 Adipose-derived stem cells (ADSCs)

The delivery of ADSCs for diabetic foot therapy requires careful consideration of injection technique to maintain cell viability and optimize therapeutic outcomes. Tseng et al. report that ADSCs retain high viability and functional capacity when administered via 27–30-gauge needles at controlled injection rates of 4 mL/min or less, minimizing mechanical stress on the cells (Tseng et al., 2024). The study also emphasizes the importance of precise administration techniques and the potential use of carrier materials to improve cell retention at the injection site, highlighting how careful handling directly impacts the efficacy of ADSC-based therapies for diabetic foot ulcers.

### 4.2 Hematopoietic stem cells (HSCs)

There are several methods for dissociating tissues in lab settings. Among the mechanical methods is the use of needles with smaller gauges, which allows separation of cells while maintaining cell viability (Ren et al., 2025). In a preclinical study, Ng et al. mechanically separated cells by using a 21G needle with a 3 mL syringe. They also used a 25G needle with 3 mL syringe to remove HSCs from multiple organs of mice to produce single-cell suspensions (Ng et al., 2025).

### 4.3 Induced pluripotent stem cells (iPSCs)

Human iPSCs can be differentiated into insulin target cells like hepatocytes, skeletal muscle cells, and adipocytes for disease modeling and therapeutic research (Thiab et al., 2025). As these iPSC-derived cells move toward injectable therapeutic applications, careful optimization of delivery parameters such as needle gauge and injection rate becomes important to preserve cell viability and maintain post-injection functionality.

Passaging hiPSCs at the appropriate cell density and timing is essential for successful cell culture. Regular monitoring of cell morphology and confluence is required. As each hiPSC line may display a unique growth rate, the split ratio should be adjusted at each passage based on colony appearance and the time required to reach 70%–80% confluence (Cheng et al., 2023). Cheng et al. in their protocol mention needle size as a probable cause of a lack of teratoma growth 3 months after injection. This is because a smaller size may cause apoptosis, and a larger size may cause an outflow from the site of administration.

### 4.4 Neural stem cells

Neural stem cell (NSC) transplantation is a practical and safe surgical approach for spinal cord injury, with peri-lesional intramedullary injections improving graft survival and host integration. Subacute transplantation may enhance cell survival and circuit formation, while chronic transplantation provides stable lesion conditions. Urine-derived induced NSCs offer a patient-specific and ethically favorable source. Precision delivery using small-gauge needles (27–30 G) and controlled infusion is essential for effective transplantation (Kwon et al., 2025).

## 5 Interaction with cell medium

Awonusi et al. found that needle gauge (22G, 23G, 27G) had minimal impact on cell viability when autologous muscle-derived cells (AMDCs) were injected at 1 × 10^7^ cells/mL. Cells suspended in type I collagen maintained near 100% viability even through smaller needles, while those in PBS showed progressive loss over 24–48 h (Awonusi et al., 2023). This suggests that at this concentration, cell survival is more dependent on the delivery vehicle than needle size, and collagen can protect cells from shear stress in narrow-bore needles.

## 6 Mechanism of length effects

### 6.1 Cumulative shear exposure

Mechanical forces encountered during cell delivery can be managed through appropriate needle and injection design to preserve stem cell viability. Mand et al. (2024) demonstrated that human ADMSCs exposed to high shear stress remained viable when shear exposure was limited in duration, while prolonged exposure led to increased cell damage. This finding supports the importance of controlling cumulative shear exposure during injection. The study also showed that the surrounding fluid environment influenced cellular tolerance to shear stress, highlighting how delivery parameters can be adjusted to protect cells. Together, these results suggest that when needles are properly selected and injection conditions are optimized, they can safely deliver stem cells while maintaining high viability.

### 6.2 Flow instabilities

In the study by Awonusi et al. (2023), flow conditions during cell injection were assessed using shear stress and Reynolds number across different needle gauges and lengths, with all configurations exhibiting Reynolds numbers well below 2,300, indicating laminar rather than unstable flow during ejection. Across 22G–27G needles and common injection vehicles, flow remained orderly with minimal mixing, indicating that classical flow instabilities such as turbulence were not induced, even under higher pressure conditions. Although smaller-gauge needles and more viscous vehicles increased shear stress, these forces occurred within a laminar regime, suggesting that for human stem cell injections, needle geometry primarily alters mechanical stress exposure rather than triggering flow instabilities that would compromise cell viability.

### 6.3 Cell aggregation effects

Human pluripotent stem cell-derived cardiomyocytes (hPSC-CMs) are delivered as single cells or 3D microtissues, with larger clusters more prone to aggregation during injection (Bain et al., 2026). Needle design and bore size can affect cell dispersion, causing backflow or uneven distribution. Specialized multi-needle systems and spheroid-compatible syringes help reduce aggregation and improve uniform cell deposition, showing that needle geometry directly influences cell clumping and retention.

### 6.4 Independent effects of flow rate

Kumar et al. conducted a study to improve the encapsulation of MSCs. They compared different flow rates and pressure values of the encapsulated cells in unpolymerized alginate to needle gauges (Kumar et al., 2022). When the flow rate was constant, pressure increased over time, especially over longer periods, which could lead to tubing connections failing. This was not noticed when the pressure was constant. The researchers noticed a higher flow rate when the diameter of the needle was bigger, and the pressure was constant. An inverse relationship was noticed between flow rates and needle length. This was attributed to greater resistance with longer needle lengths. These findings highlight that careful optimization of injection parameters, including needle characteristics and flow rate, is critical for maintaining stem cell integrity and ensuring effective clinical outcomes, suggesting that gauge and length may similarly influence cell retention and functionality during transplantation.

## 7 Optimization strategies

Optimizing injection parameters is critical for maintaining the viability of human stem cells during transplantation. Paulsen et al. demonstrated that clinical-grade hiPSC-derived ovarian support cells maintained high viability (>90%) across multiple handling and storage conditions, highlighting the importance of carefully controlled processing to preserve cellular integrity (Paulsen et al., 2026). Although their study focused on *in vitro* handling, these findings suggest that minimizing mechanical stress during injection, through appropriate needle gauge, length, and flow rate, could similarly protect cells during delivery. This directly relates to needle gauge and length, as selecting the proper needle dimensions can reduce shear stress and improve cell survival during injection. Therefore, optimization strategies that reduce shear forces and limit prolonged exposure to harsh conditions are essential for ensuring the efficacy of stem cell therapies.

### 7.1 Syringe injection vs. 3D bioprinting: a comparative perspective on cell viability

Syringe-based injections and extrusion-based 3D bioprinting share a fundamental mechanical challenge where cells must be forced through a narrow orifice under pressure. This exposes them to shear and compressive forces that can compromise viability. However, the two modalities differ substantially in their control over these parameters, and comparing them is instructive for optimizing stem cell delivery.

In conventional syringe injections, shear stress is governed primarily by needle gauge, injection rate, and cell suspension viscosity. As discussed throughout this review, smaller gauge needles (e.g., 27G–30G) generate substantially higher shear stresses, and cell viability decreases proportionally. Injection is typically a rapid, single-pass event, and the operator has limited real-time control once the plunger is depressed. Cell viability post-injection using optimized syringe protocols for MSCs generally ranges from 70% to 90% with 21G–23G needles, though this can fall substantially with smaller-bore needles or faster injection rates (Aguado et al., 2012; Mand et al., 2024).

Extrusion-based 3D bioprinting subjects cells to comparable or greater mechanical stress, as bioinks are typically more viscous than simple saline or PBS suspensions and are extruded continuously through fine nozzles (often with an inner diameter of 200–400 μm, roughly equivalent to 22G–25G). Studies have reported post-printing cell viabilities ranging from 40% to over 90%, depending on nozzle diameter, print speed, bioink composition, and cell density (Gudapati et al., 2016). Critically, 3D bioprinting allows programmable, consistent control of flow rate and pressure throughout the deposition process. With syringe injections, these parameters depend on operator technique and syringe compliance. This consistency can translate to more reproducible viability outcomes across batches.

A key differentiating factor is the role of the carrier material. In 3D bioprinting, cells are encapsulated within hydrogel bioinks (e.g., gelatin methacryloyl, alginate, or fibrin-based formulations) that provide viscoelastic buffering, reducing the effective shear stress experienced at the cell membrane. This is analogous to the protective effect of type I collagen carriers observed in syringe injection studies (Awonusi et al., 2023). In contrast, cells in syringe injection are frequently suspended in physiological saline or PBS, offering minimal mechanical protection.

The trade-off is between precision and simplicity. Syringe injection is portable, rapid, and clinically feasible in a broad range of settings. 3D bioprinting requires specialized equipment, sterile printing environments, and optimized bioink formulations. For *in vivo* stem cell delivery to anatomically accessible sites (e.g., intra-articular, intradermal, or intramuscular), syringe injection with optimized parameters remains the standard of care. Bioprinting is more relevant for the *ex vivo* fabrication of tissue constructs intended for implantation, where precise spatial deposition of cells is more important than speed. Table 1 (below) summarizes key comparative parameters.

**TABLE 1:** Comparative parameters of syringe injection and extrusion-based 3D bioprinting for stem cell delivery.

Parameter	Syringe injection	Extrusion-based 3D bioprinting
Typical orifice diameter	210–840 µm (30G–21G)	150–600 µm (nozzle-dependent)
Carrier medium	PBS, saline, collagen	Hydrogel bioink (alginate, GelMA, fibrin)
Shear stress range	10–1,000+ Pa (gauge-dependent)	50–500 Pa (nozzle/speed-dependent)
Reported MSC viability	70%–95% (optimized)	40%–95% (bioink/parameter-dependent)
Flow control	Operator-dependent	Programmable, reproducible
Clinical feasibility	High	Low (requires specialized equipment)
Primary application	*In vivo* cell delivery	*Ex vivo* tissue construct fabrication
Cell protection mechanism	Delivery vehicle viscosity	Bioink viscoelasticity and encapsulation

The evidence suggests that the fundamental fluid-mechanical principles governing cell damage during syringe injection, i.e., shear stress as a function of orifice radius and flow rate, apply equally to nozzles used in 3D bioprinting. Optimization strategies developed for one modality, such as reducing flow rate, increasing carrier viscosity, or reducing orifice constriction, translate directly to the other. Future research comparing these two delivery modes head-to-head with standardized cell types, carrier formulations, and viability assays would substantially advance the field.

## 8 Clinical implementation

Clinical implementation should ensure that all users are trained on their specific syringe pump system to prevent infusion errors and improve patient safety. Weiss et al. observed that using the smallest appropriate Luer-lock syringe reduces start-up delays, flow irregularities, and bolus release during line occlusion, thereby improving the accuracy of administered solutions (Weiss et al., 2023). Additionally, minimizing the compliance and resistance of the syringe pump system and avoiding very low flow rates helps ensure consistent delivery. Also, adherence to standardized protocols for pump handling is critical to prevent errors and maintain reliable infusion.

### 8.1 Orthopedic clinical applications

#### 8.1.1 Intra-articular injection

Rahmadian et al. conducted a meta-analysis of six randomized controlled trials, including eight MSC treatment arms, and found that intra-articular injections significantly improved WOMAC scores at 12 months (Rahmadian et al., 2025). Their analysis showed that MSC doses of ≤25 million cells produced statistically significant improvements, whereas higher doses did not confer additional benefit, suggesting that lower doses may be sufficient for clinical efficacy. The study also highlighted moderate heterogeneity (*I* ^*2*^ = 49.8%), reflecting variability in MSC sources, doses, and patient populations across trials. These findings support the importance of optimizing MSC dosing and may help explain why factors such as cell viability at the time of injection could influence treatment outcomes.

Many clinical trials often administer MSCs via intra-articular injection using 20G needles, as replicated by Lee et al. to increase the number of viable cells received by the patient; this may cause more discomfort compared to smaller gauge needles (Lee et al., 2025). However, the biologics may still experience some mechanical shear stress when a denser cell suspension is administered. This may affect cell integrity during cell administration. These findings highlight the importance of optimizing MSC dosing and may help explain why factors such as cell viability at the time of injection could influence treatment outcomes.

#### 8.1.2 Intratendinous injection

In a randomized controlled trial investigating intralesional MSC injections for partial-thickness supraspinatus tendon tears, all participants experienced only transient pain at the injection site, with no persistent adverse events reported, indicating that the injection process was generally safe (Chun et al., 2022). Despite careful administration, MSC injection did not result in superior improvements in pain or shoulder function compared with control treatments, suggesting that mechanical factors such as needle penetration and injection technique could influence the efficacy of stem cell delivery.

Moreover, regenerative tendon studies utilize 26–29G needles to deliver tdECM hydrogels and stem cell–derived exosomes, which lead to sustained release and improvements in tissue structure, biomechanics, and function (Li et al., 2025). These results highlight that while intratendinous injections can be performed safely, optimizing needle gauge, length, and delivery method may be critical to preserve stem cell integrity and maximize regenerative potential.

#### 8.1.3 Bone injection (subchondral bone and fracture sites)

Subchondral injections of MSCs are increasingly explored as a treatment for bone marrow lesions, including osteoarthritis and osteochondral abnormalities. These injections deliver MSCs directly into affected bone, providing a microenvironment for cartilage and bone repair while potentially reducing pain and improving function (Pasculli et al., 2022). A preclinical study using human MSCs administered subchondral injections through 26G and 29G needles to reduce weakening of the trabecular mesh (Wu et al., 2025).

#### 8.1.4 Intervertebral disc injection

Bone marrow-derived mesenchymal stem cells (BMSCs) can promote intervertebral disc repair by increasing nucleus pulposus (NP) cell proliferation, enhancing extracellular matrix components like collagen and proteoglycans, and improving cell survival in degenerative disc models. *In vivo* studies demonstrated that injected BMSCs survive, migrate to injured areas, and restore disc height and tissue structure, confirming their functional integration into degenerative discs (Zhang et al., 2022).

Cervical and thoracic injections typically utilize needles of 21G-22G and a length of 4–8 cm. In contrast, lumbar injections require needles of 20G-21G and a length of 7–12 cm (Bogdanovic et al., 2023). In clinical practice, intradiscal stem cell therapy typically uses thin spinal needles (like 22G) guided by imaging to deliver MSCs. Since the needle size and how deep it goes can affect cellular survival, understanding and reporting these details is important to help ensure the stem cells remain healthy and the treatment works as well as possible (Maal et al., 2025).

### 8.2 Neurological clinical applications

#### 8.2.1 Intrathecal administration

Intrathecal administration of human UC-MSCs has been shown to effectively alleviate bone cancer pain while maintaining a favorable safety profile. In preclinical rat models, careful use of a 50 µL Hamilton syringe and appropriate needle insertion at the L4-L5 intervertebral space allowed for precise delivery of 1 × 10^6^ or 4 × 10^6^ hUC-MSCs without significant leakage or cell loss, demonstrating that mechanical stress during intrathecal injection can be minimized with proper technique (Zhang et al., 2025).

#### 8.2.2 Intracerebral injection (surgical or experimental)

In a phase I clinical trial investigating secondary progressive multiple sclerosis, allogeneic human neural stem/progenitor cells (hNSCs) were delivered via intracerebroventricular injection (ICVI). No treatment-related deaths or SAEs were observed over a 12-month follow-up, indicating that the procedure preserved overall cell tolerability and safety in humans (Leone et al., 2023). The recovered cells from the injection needle in a subset of the cases retained normal growth and differentiation capacity after expansion.

This suggests that the mechanical forces encountered during ICVI delivery did not significantly compromise stem cell viability. For their preclinical study, Leone et al. used 30G, 1 cm long, point style 4 45° and observed similar results. Based on these findings, it can be inferred that the use of appropriately sized needle gauges and lengths in intracerebral delivery may minimize shear stress and mechanical damage, thereby supporting higher post-injection stem cell viability and functional integrity.

#### 8.2.3 Peripheral nerve injection

Stem cell therapies have been locally delivered to peripheral nerve injury sites to support axonal regeneration and functional recovery (Wynne et al., 2024). Their routes of administration include the use of specialized scaffolds, microinjections for precision, or intravenous or intrathecal injection (Widodo et al., 2025). These regenerative effects are largely attributed to the survival of transplanted cells and to paracrine signaling mechanisms. Therefore, during peripheral nerve injection, injection parameters such as needle gauge and length may influence therapeutic outcomes by affecting cell viability during delivery.

### 8.3 Cardiovascular clinical applications

#### 8.3.1 Intramyocardial injection

Intramyocardial injection of Human UCMSCs using a 30-gauge needle allowed transplanted cells to survive in murine myocardium for at least 28 days and significantly improved cardiac function after myocardial infarction (Liu et al., 2022). This localized delivery also enhanced angiogenesis and reduced cardiac fibrosis and hypertrophy, indicating that viable cells were successfully retained at the injection site following administration. Based on these findings, needle gauge and length during intramyocardial injection may influence stem cell viability by altering mechanical stress and retention of cells within dense myocardial tissue.

#### 8.3.2 Intracoronary infusion

Transendocardial injection is another route of administration that uses needle catheters for stem cell therapy for advanced heart disease (Raval and Pepine, 2021). Some commonly used needles for these procedures range from 25G to 27G. In their study, the researchers observed the safety of different needle designs (helical, electro-anatomically tracked straight needle, straight, and curved) and found the helical design to perform significantly better than the others. It can be inferred that needle shape is as important a consideration as is the needle size.

### 8.4 Aesthetic and reconstructive applications

#### 8.4.1 Subcutaneous injection

Subcutaneous injection of MSCs using a 25-gauge needle delivers 5 × 10^6^ live cells in 200 μL of saline, but this method results in a less dense local capillary network than implantation of an MSC pellet, suggesting potential cell loss or reduced viability during injection (Mammoto et al., 2026). Implanted MSC pellets, in contrast, maintain cell–cell interactions and extracellular matrix formation, supporting stable engraftment and sustained secretion of angiogenesis-related factors such as FGF-1 and FGF-2, which are detectable in recipient mouse plasma. These findings suggest that needle gauge and injection length may mechanically stress MSCs during delivery, potentially reducing viability and angiogenic potential compared with pellet implantation.

#### 8.4.2 Intradermal injection

Intradermal injection using an automated perpendicular multi-needle device allows precise delivery of MSCs into the dermis, with needle penetration above 1.0 mm achieving delivery from up to 89% of needles and minimal reflux (Rangatchew et al., 2023). Post-ejection analysis demonstrated that while short-term cell viability, adherence, metabolism, and IDO1-expression were largely unaffected, long-term viability and proliferation decreased slightly, indicating stress on cells during needle passage. Therefore, selecting an appropriate needle gauge and configuration, such as the 9-pin 31G head, may help minimize cellular stress while maintaining effective dermal delivery (inference).

## 9 Limitations

Across all applications reviewed, a consistent pattern emerged. Published studies rarely report needle gauge and length as primary outcome variables, and viability measurements taken after injection are frequently absent or limited to pre-injection aliquots. This represents a significant methodological gap, as the mechanical stress experienced during transit is fundamentally distinct from that measured in a static culture well. Table 2 (below) highlights that in the majority of clinical and preclinical studies, needle parameters are reported incidentally rather than systematically investigated, and that sample sizes are generally insufficient to draw conclusions about gauge-specific viability thresholds in the clinical setting.

**TABLE 2:** Summary of needle gauge and length effects on human stem cell viability across clinical applications: key findings, limitations, and operator-dependent factors.

Application/Cell type	Optimal needle parameters (reported)	Key viability finding	Study design limitation	Operator-dependent factors	Reference(s)
ADSCs — diabetic foot	27G–30G; ≤4 mL/min	High viability and functional capacity retained at controlled injection rates	Single-centre; limited long-term functional follow-up of injected cells	Injection rate consistency; depth of subcutaneous placement; manual pressure on plunger	Tseng et al. (2024)
MSCs — intra-articular (knee OA)	20G; standard length	Viable cell delivery achieved; lower doses (≤25 × 10^6^) sufficient for efficacy	High inter-trial heterogeneity (I^2^ = 49.8%); MSC source variability across included trials	Intra-articular landmark accuracy; use of ultrasound guidance vs. landmark-only technique; aspiration before injection	Rahmadian et al. (2025), Lee et al. (2025)
MSCs — intratendinous	26G–29G	Safe delivery; no persistent adverse events; no superior functional outcome vs. control	Single RCT; mechanical factors of needle penetration not standardized across operators	Needle angle of approach; tendon depth estimation; degree of needle fenestration of tissue	Chun et al. (2022), Li et al. (2025)
MSCs — subchondral bone	26G; 29G	Reduced trabecular weakening in preclinical model	Preclinical only (human MSCs in animal model); no clinical viability data post-injection	Bone cortex resistance variability; stylet use; image-guided vs. freehand placement	Wu et al. (2025), Pasculli et al. (2022)
BMSCs — intradiscal	22G; 4–8 cm (cervical/thoracic); 20G–21G, 7–12 cm (lumbar)	Functional integration demonstrated *in vivo*; disc height restoration	Lack of standardized reporting of needle gauge and insertion depth across published trials	Fluoroscopic vs. CT guidance; operator experience level; patient BMI affecting needle depth estimation	Bogdanovic et al. (2023), Zhang et al. (2022), Maal et al. (2025)
hUC-MSCs — intrathecal	50 µL Hamilton syringe; L4–L5 intervertebral insertion	Precise delivery without significant leakage or cell loss when proper technique applied	Preclinical rat model; extrapolation to human anatomy and tissue resistance is uncertain	Spinal level identification accuracy; rate of intrathecal bolus delivery; patient positioning	Zhang et al. (2025)
hNSCs — intracerebral (ICV)	30G; 1 cm; 45° point style	Normal growth and differentiation capacity retained post-delivery in recovered cells	Phase I trial (n small); viability measured only in a subset of recovered needle-residual cells, not in implanted population	Stereotactic frame calibration; neurosurgeon experience; angle of approach to ventricle	Leone et al. (2023)
hUC-MSCs — intramyocardial	30G	Cell survival ≥28 days; improved cardiac function; reduced fibrosis	Murine model; myocardial tissue density differs from human; injection volume per site not standardized	Epicardial vs. transendocardial route; catheter stability during cardiac cycle; echocardiographic guidance quality	Liu et al. (2022)
MSCs — transendocardial (catheter)	25G–27G; helical needle design	Helical design outperformed straight, curved, and electro-anatomically tracked designs for cell retention	Non-randomized safety study; viability not directly quantified post-injection	Catheter navigation skill; fluoroscopic vs. electro-anatomic mapping guidance; contact force sensing	Raval and Pepine (2021)
MSCs — subcutaneous	25G	Reduced capillary network density vs. pellet implantation; probable cell loss during injection	No direct intra-needle viability measurement; comparative arm (pellet) not deliverable *via* standard injection	Subcutaneous depth control; bolus vs. depot technique; post-injection site compression	Mammoto et al. (2026)
MSCs — intradermal (multi-needle device)	31G; 9-pin automated perpendicular device	Short-term viability largely preserved; long-term viability and proliferation slightly decreased	Automated device not universally available; limited data on freehand intradermal technique at equivalent gauge	Device calibration; needle penetration depth setting; skin tension and hydration at time of injection	Rangatchew et al. (2023)
AMDCs — laryngeal injection	22G–27G in collagen carrier	Near 100% viability across all gauges tested in collagen; progressive viability loss in PBS at 24–48 h	Single cell type and injection vehicle pairing; results may not generalize to other cell types or carriers	Carrier material selection and preparation; temperature of cell suspension at time of injection; suspension homogeneity	Awonusi et al. (2023)
hiPSC-derived cells — general	Gauge selection case-specific	Smaller gauge associated with apoptosis; larger gauge associated with outflow from administration site	Protocol-level guidance only; no systematic human viability data across gauge range	Passage number and cell density at time of injection; split ratio consistency; colony confluence assessment	Cheng et al. (2023)
HSCs — mechanical dissociation	21G (tissue dissociation); 25G (single-cell suspension harvest)	Cell viability maintained with smaller gauges when used for controlled mechanical separation	Preclinical (murine) tissue source; needle use here is for dissociation, not therapeutic delivery	Number of needle passes; syringe volume; speed of aspiration and ejection strokes	Ng et al. (2025)
NSCs — spinal cord (intramedullary)	27G–30G; controlled infusion rate	Peri-lesional intramedullary delivery supports graft survival and host integration	Timing of transplantation (subacute vs. chronic) affects outcome independently of needle parameters	Lesion localization accuracy; infusion pump rate calibration; dural entry technique	Kwon et al. (2025)
MSCs — syringe injection vs. 3D bioprinting (comparative)	Syringe: 21G–23G optimal; bioprinting: 200–400 µm nozzle	Syringe viability 70%–95% (optimized); bioprinting viability 40%–95% (bioink-dependent)	No head-to-head RCT comparing modalities with standardized cell type and carrier; heterogeneous outcome reporting	Syringe: Plunger pressure consistency, injection rate, needle priming; bioprinting: Nozzle temperature, print speed, bioink gelation timing	Aguado et al. (2012), Gudapati et al. (2016), Mand et al. (2024), Kumar et al. (2023)

Operator-dependent factors represent a particularly underappreciated source of variability. Injection rate, which is arguably the single most influential modifiable variable after gauge selection, is rarely standardized or reported in clinical protocols. This is despite strong *in vitro* evidence that slower rates substantially mitigate shear stress at any given gauge (Mand et al., 2024; Aguado et al., 2012). Similarly, the temperature of the cell suspension at time of delivery, the degree of pre-injection agitation (which affects cell aggregation state), and the use of image guidance versus anatomical landmark technique all introduce variability in outcomes that is attributable to the operator rather than the needle itself. Training standardization, use of syringe pump systems for controlled infusion, and mandatory pre- and post-injection viability testing represent achievable near-term improvements to clinical protocols that would substantially reduce this operator-dependent variability (Weiss et al., 2023). Until such standards are adopted and consistently reported in clinical trials, meta-analytic comparisons of delivery outcomes across studies will remain limited by confounding due to differences in administration technique.

## 10 Future directions and emerging technologies

### 10.1 Microfluidic injection systems

**FIGURE 2:**
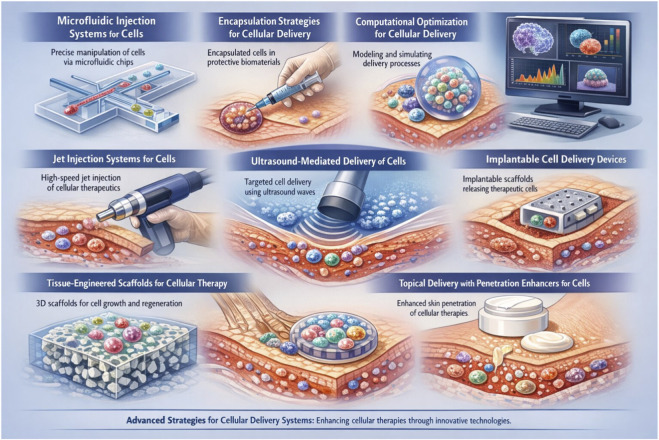
Comprehensive overview of engineering and biophysical approaches for targeted cellular delivery in regenerative medicine and therapeutic applications. The figure illustrates eight major technological platforms designed to enhance the precision, viability, targeting efficiency, and therapeutic performance of delivered cells.

Microfluidic injection systems use precisely engineered networks of miniaturized channels, typically 10–500 µm in size, to manipulate small volumes of fluids and suspended cells with high control (Bendre et al., 2022). Microfluidic devices offer several advantages over conventional syringe injection. The flow rates and pressure profiles can be controlled with far greater precision, reducing peak shear stress exposure during cell transit. Microfluidic platforms can generate monodisperse droplets or microgels on-chip, enabling real-time encapsulation of cells immediately prior to injection, minimizing the window during which cells are exposed to mechanical stress without a protective carrier (Figure 2). Organ-on-chip systems, a subclass of microfluidic devices that incorporate living cell cultures within their channel architecture, enable the study of interactions between injected cells and target tissue environments under physiologically relevant flow conditions before clinical application. Current limitations include the need for specialized microfabrication infrastructure, potential for channel blockage at high cell densities, and challenges in scaling throughput to volumes required for clinical-grade cell doses.

### 10.2 Encapsulation strategies

Encapsulation involves surrounding individual cells or small clusters of cells with a biocompatible shell or matrix prior to injection, to physically shield the cell membrane from shear and compressive forces during needle transit (Kaur et al., 2026). The most widely studied encapsulation platforms include alginate microbeads (produced by ionic crosslinking in a calcium chloride bath), gelatin methacryloyl (GelMA) microgels (photo-crosslinked under UV light), and fibrin-based matrices. Microfluidic droplet generation is increasingly used to produce encapsulation capsules with tightly controlled diameters (typically 50–500 µm), high monodispersity, and thin shells that permit diffusion of oxygen, nutrients, and secreted paracrine factors while maintaining mechanical integrity under shear. Kumar et al. demonstrated that MSCs encapsulated in unpolymerized alginate prior to needle passage maintained higher viability than cells in suspension, and that encapsulation parameters, including capsule diameter and alginate concentration, interacted with needle gauge to determine cell survival (Kumar et al., 2022). Beyond mechanical protection, encapsulation can also improve cell retention at the injection site by preventing passive dispersal, and can provide an immunoprotective barrier in allogeneic transplantations. Key challenges include ensuring adequate capsule degradation or resorption post-injection to enable cell engraftment, preventing capsule aggregation in narrow-bore needles, and scaling encapsulation workflows to clinical cell numbers.

### 10.3 Computational optimization

Computational optimization uses mathematical models, simulation tools, and machine learning algorithms to predict and minimize the mechanical stress cells experience during injection, without requiring exhaustive empirical testing of every parameter combination (Yang et al., 2022). The computational fluid dynamics (CFD) modeling solves Navier–Stokes equations governing viscous fluid flow numerically within three-dimensional models of needle geometries. CFD simulations can generate spatial maps of shear stress, velocity gradients, and pressure distribution throughout the needle lumen, identifying zones of peak mechanical stress and predicting how changes in gauge, length, flow rate, or carrier viscosity will affect the distribution of forces on suspended cells. The second approach involves data-driven machine learning models, including Gaussian process regression, neural networks, and random forests. These are trained on experimental datasets mapping injection parameters to viability outcomes. These models can identify non-obvious interactions between variables (for example, the way that cell density modulates the protective effect of a viscous carrier) and can propose optimized parameter combinations that a purely theoretical approach might miss. Importantly, computational approaches can be extended to model specific anatomical injection sites, incorporating tissue resistance and backpressure into the optimization. Integration of CFD simulation with clinical trial outcome data represents an emerging and potentially powerful approach for prospectively designing injection protocols that maximize viable cell delivery.

### 10.4 Jet injection systems

Jet injection systems propel a liquid stream at high velocity through a small orifice, penetrating tissue without a conventional hollow-bore needle. Needle-free jet injectors and bead-jet printing platforms offer potential advantages for cellular therapy applications where minimizing tissue trauma or achieving precise spatial deposition of cells is a priority (Cao et al., 2024). In bead-jet printing, individual droplets containing cells are generated by acoustic or piezoelectric actuation and deposited with micrometer-level positional accuracy. This modality is particularly relevant for skin regeneration, wound bed seeding, and ocular applications. Cell viability in jet-injected or jet-printed MSC preparations has been reported to be high when ejection parameters are optimized, with the critical variables being droplet generation frequency, jet velocity, and cell concentration. At high frequencies or velocities, cells experience extensional stresses during droplet breakoff that can exceed membrane rupture thresholds; careful titration of these parameters is therefore necessary. A key advantage of jet systems is their ability to deliver cells in sparse, uniform patterns with high reproducibility, avoiding the cell aggregation and local concentration peaks that can occur with bolus syringe injection.

### 10.5 Ultrasound-mediated delivery

Ultrasound-mediated delivery uses focused acoustic energy to transiently increase tissue permeability or to actively guide cell-laden constructs to target sites (Arrieta et al., 2024). In sonoporation, high-intensity focused ultrasound (HIFU) or low-frequency ultrasound, combined with microbubble contrast agents, creates transient pores in cell membranes and endothelial barriers, thereby enhancing the local uptake and retention of systemically delivered cells. In acoustic radiation force-based approaches, the mechanical momentum of an ultrasound beam is used to push cells toward a target tissue surface, improving engraftment efficiency after intravenous or intravascular infusion. For intraparenchymal delivery, ultrasound guidance improves needle placement accuracy and can be used to monitor the distribution of injected cell suspensions in real time. The ultrasound parameters required for tissue permeabilization (particularly peak negative pressure and mechanical index) must be carefully controlled to avoid direct acoustic damage to cells, including membrane disruption from microbubble cavitation. Current clinical translation is most advanced in central nervous system applications, where focused ultrasound-mediated opening of the blood–brain barrier is being actively investigated as a route for delivering cellular therapeutics.

### 10.6 Implantable cell delivery devices

Implantable cell delivery devices are engineered constructs, typically fabricated from biocompatible polymers or hydrogels, that are surgically or minimally invasively placed at a target anatomical site and then slowly release encapsulated cells over a defined time course (Huanbutta et al., 2024). Unlike single-bolus injection, implantable devices maintain a protected microenvironment for cells, shielding them from host immune responses and providing sustained local delivery of secreted factors. Designs range from macroscopic scaffolds and hydrogel depots implanted through small-bore catheters or trocars, to microscale devices that can be injected through needles as large as 16G–18G before expanding *in situ* to their final geometry. Needle selection for device deployment involves different design considerations than for cell suspension injection. The primary concern in this case is not shear-induced cell death during transit but rather the integrity of the device architecture (including pore structure and membrane thickness) and its ability to support cell viability within the implant over weeks to months. Key parameters include oxygen and nutrient diffusion distance (typically limited to 150–200 µm from the nearest vasculature), the immune barrier function of the device membrane, and the degradation kinetics of the biomaterial scaffold.

### 10.7 Tissue-engineered scaffolds

Tissue-engineered scaffolds are three-dimensional porous structures, fabricated from natural or synthetic biomaterials, that provide a physical substrate for cell attachment, proliferation, and differentiation prior to or following implantation (Liu et al., 2024). These scaffolds serve as an alternative to suspension-based injection by maintaining cell-to-cell and cell-to-matrix interactions that are disrupted when cells are dissociated for syringe delivery. Scaffold geometry, specifically pore size, interconnectivity, and surface topography, has been shown to directly influence cell alignment, differentiation trajectory, and secretome profile. Oriented porous scaffolds, in which pores are aligned along a defined axis, are particularly effective at directing cell growth and supporting aligned tissue formation in applications such as tendon repair, spinal cord regeneration, and myocardial tissue engineering. Unlike needle-injected cells, scaffold-delivered cells are not exposed to transit shear stress. However, the scaffold itself must often be delivered via a minimally invasive route (e.g., an arthroscopic cannula or a laparoscopic port). This introduces considerations around structural integrity during deployment. Injectable scaffold precursors, such as bioinks or hydrogel precursor solutions that crosslink *in situ* after injection, represent a convergence point between scaffold-based and syringe-based delivery. These combine the mechanical protection of a matrix environment with the minimally invasive access offered by needle delivery.

### 10.8 Topical delivery with penetration enhancers

Topical delivery with penetration enhancers encompasses strategies that bypass the needle entirely by facilitating the trans-epithelial or transmucosal transport of cells or cell-derived therapeutic factors across biological barriers. Cell-penetrating peptides (CPPs) are short amino acid sequences (typically 5–30 residues) that interact with negatively charged plasma membrane phospholipids via electrostatic interactions. This enables intracellular transport of attached cargo via mechanisms such as macropinocytosis, clathrin-mediated endocytosis, and direct membrane translocation. CPPs are most commonly used to deliver cell-derived exosomes, growth factors, nucleic acids, or small molecules that recapitulate the paracrine effects of stem cells without requiring the administration of viable cells. Intact cells are too large for transepithelial transport (Wu et al., 2024). Physical penetration enhancers, including microneedle arrays, electroporation, sonophoresis, and iontophoresis, can also be used in combination with cell-derived therapeutics to enhance dermal or mucosal delivery. Microneedle-based delivery of MSC-derived exosomes has attracted particular attention for wound healing, hair restoration, and skin rejuvenation applications, where painless and precise epidermal targeting is desirable. The primary advantage of these approaches, from a viability standpoint, is the complete elimination of transit shear stress because no intact cells pass through the needle lumen. The principal limitation is that the full spectrum of cell-mediated repair mechanisms, including direct engraftment, cell–cell signaling, and immunomodulation by transplanted cells, cannot be replicated by acellular or exosome-based formulations alone.

## 11 Regulatory documentation

Comprehensive documentation of injection parameters should include needle gauge and length specifications with manufacturer identification, injection rate (measured or estimated) or total injection time, cell concentration and total volume administered, cell viability pre-injection and post-injection (when measured), operator identification for quality monitoring and training assessment, any deviations from standard protocols with justification and adverse events occurring during or immediately after injection. This documentation enables continuous quality improvement, supports regulatory compliance, facilitates comparison across clinical trials, and provides evidence for protocol refinement.

## 12 Conclusion

The viability of human stem cells during injection can be influenced by needle gauge, with smaller needles producing progressively greater cell damage through increased shear stress. The relationship follows theoretical predictions from fluid mechanics, with shear stress increasing proportionally to the inverse cube of needle radius. Needle length exerts a secondary but measurable effect, particularly for long needles in small-bore configurations. The interaction between these physical parameters and injection rate is critical, with slower injection rates partially mitigating the damage from smaller needles.

For MSCs, the most commonly used stem cell type in clinical applications, needles in the 21G-23G range with standard lengths (25–40 mm) and moderate injection rates (0.2–0.4 mL/s) provide optimal balance between cell preservation (>85% viability), tissue access, and clinical practicality. Smaller needles can be used when minimizing tissue trauma is a primary concern, but require proportionally slower injection rates and may still result in 15%–30% viability loss with needles below 27G, even with optimized technique.

Clinical protocols should be customized for each specific application, considering the anatomical requirements, cell type sensitivity, therapeutic cell dose requirements, and acceptable viability thresholds. The evidence clearly demonstrates that conventional approaches are suboptimal, and practitioners should select needle parameters based on the specific clinical context, while being aware of the trade-offs involved.

Future advances in delivery technology, including microfluidic systems, protective encapsulation strategies, and computational optimization tools, may enable the use of smaller needles without compromising therapeutic efficacy. Alternative delivery methods eliminating needle passage entirely show promise for specific applications. However, current best practice favors the largest practical needle gauge to maximize viable cell delivery, supplemented by careful attention to injection rate, needle length, and cell-suspension optimization.

The field would benefit substantially from standardized reporting of injection parameters in clinical trials, allowing meta-analysis of outcomes relative to delivery methodology. As stem cell therapies continue to advance toward widespread clinical implementation, optimization of administration techniques represents a readily addressable factor in improving therapeutic outcomes. Given that injection-related cell loss can approach more than 50% with suboptimal parameters, attention to these technical details may significantly impact treatment success.
